# Genetic analyses of early-onset Alzheimer’s disease using next generation sequencing

**DOI:** 10.1038/s41598-019-44848-2

**Published:** 2019-06-10

**Authors:** Vo Van Giau, Eva Bagyinszky, Young Soon Yang, Young Chul Youn, Seong Soo A. An, Sang Yun Kim

**Affiliations:** 1 Department of Bionano Technology, Gachon University, Seongnam, 13120 South Korea; 2 Department of Neurology, Veterans Health Service Medical Center, Seoul, 05368 South Korea; 3 Department of Neurology, Chung-Ang University Hospital, Seoul, 06973 South Korea; 4 Department of Neurology, Seoul National University College of Medicine & Neurocognitive Behavior Center, Seoul National University Bundang Hospital, Seongnam, 13620 South Korea

**Keywords:** Next-generation sequencing, Developmental neurogenesis

## Abstract

Alzheimer’s disease (AD) is the most common type of neurodegenerative dementia, but the cause of AD remained poorly understood. Many mutations in the amyloid precursor protein (*APP*) and presenilin 1 and 2 (*PSEN1* and *PSEN2*) have been reported as the pathogenic causes of early-onset AD (EOAD), which accounts for up to 5% of all AD cases. In this study, we screened familiar/*de novo* EOAD (n = 67) samples by next-generation sequencing (NGS) of a 50-gene panel, which included causative and possible pathogenic variants linked to neurodegenerative disorders. Remarkably, three missense mutations in *PSEN1* (T119I, G209A, and G417A) and one known variant in *PSEN2* (H169N) were discovered in 6% of the cases. Additionally, 67 missense mutations in susceptibility genes for late-onset AD were identified, which may be involved in cholesterol transport, inflammatory response, and β-amyloid modulation. We identified 70 additional novel and missense variants in other genes, such as *MAPT*, *GRN*, *CSF1R*, and *PRNP*, related to neurodegenerative diseases, which may represent overlapping clinical and neuropathological features with AD. Extensive genetic screening of Korean patients with EOAD identified multiple rare variants with potential roles in AD pathogenesis. This study suggests that individuals diagnosed with AD should be screened for other neurodegenerative disease-associated genes. Our findings expand the classic set of genes involved in neurodegenerative pathogenesis, which should be screened for in clinical trials. Main limitation of this study was the absence of functional assessment for possibly and probably pathogenic variants. Additional issues were that we could not perform studies on copy number variants, and we could not verify the segregation of mutations.

## Introduction

Alzheimer disease (AD) is a devastating neurodegenerative disease accounting for 50–75% of all forms of dementia. Approximately 44 million people worldwide were estimated to be diagnosed with AD or a related dementia in 2015^1^. Approximately 4.6 million new cases of dementia are reported annually, and the number of AD patients is expected to nearly double by 2030^1^. Genetic factors may explain many of the variations affecting AD risk, particularly familial AD and early-onset AD (EOAD), in which most genetic variants are related to amyloid-β (Aβ) processing^2–4^. EOAD is a subtype of AD in which disease onset occurs before the age of 65 years, but several patients develop AD in their 30 s or 40s^5–8^. Three genes have been identified as causative factors for EOAD: amyloid precursor protein (*APP*)^5,9^, presenilin 1 (*PSEN1*)^6–8,10^, and presenilin 2 (*PSEN2*)^11,12^. To date, >270 highly penetrant mutations (http://www.molgen.ua.ac.be/admutations/; http://www.alzforum.org/mutations) in these genes were reported to cause familial AD, and emerging studies continue to report additional novel mutations^13–15^. However, few cases of high-penetrant mutations in *APP*, *PSEN1*, and *PSEN2* can explain the causality in EOAD families (5–10%), with a large group of autosomal dominant pedigrees unexplained genetically^16–18^. Patients with late-onset AD (LOAD) are more common in people at the age of 65 years or older^15,19^. Large-scale genome-wide association studies (GWAS) identified >20 genetic loci, including *APOE*, associated with increased susceptibility to LOAD, which could be involved in the pathway of Aβ production and clearance^20,21^. The heritability of LOAD was estimated to be approximately 80%^15,22^. Thus, a better understanding of genetic susceptibilities would be essential for early detection and treatment^23–27^. Studies of the differences in heritability and age of onset between EOAD and LOAD may explain why EOAD patients have a more penetrant genetic etiology, thus, providing insight for discovering the genetic causes of AD.

Clinically, many types of dementia may have neuropathological or clinical crossover. For example, Parkinson’s disease (PD) patients both with or without dementia exhibit alpha-synuclein deposits in the brain, and patients with dementia with Lewy bodies can show similar clinical presentations^14^. Many similarities in genetic variants were reported between different neurodegenerative disorders, such as rare *TREM2* variants in AD^28^, while *TREM2* p.R47H was reported as a risk variant for PD, frontotemporal dementia (FTD), and amyotrophic lateral sclerosis (ALS)^29,30^. Remarkably, LOAD is genetically far more complex than EOAD, and some genetic loci were shown to be associated with multiple types of dementias; the most common example is APOE ε4, which was suggested to be associated with AD, PD, and other neurodegenerative diseases^21^. More interestingly, a recent study demonstrated that *PARK2* (p.T240M, p.Q34fs delAG) variants in early-onset PD and *MAPT* (p.A469T) are also associated with *de novo* EOAD^31^. Sporadic neurodegenerative diseases, such as PD, AD, multiple system atrophy, Creutzfeldt-Jakob disease (CJD), ALS, and corticobasal degeneration disease, can present similar clinical phenotypes as progressive supranuclear palsy, which may result in a false diagnosis^32,33^. These findings suggest the need for a detailed screening of patients with EOAD to improve differential diagnosis, as may harbor potential causative variants of PD and FTD.

Thus, although many risk genes have been reported for AD, the identification of additional disease-associated genes remained challenging. Recently, next-generation sequencing (NGS) technologies have been used to examine patients with EOAD patients with unknown etiology^34^, NGS may provide fast and cost-effective sequencing strategies and sequence an entire genome in >1 day. In this study, an NGS panel of 50 genes described previously^19^ was used to evaluated 67 Korean patients with EOAD.

## Results

### *PSEN1* and *PSEN2* variants

**Figure 1:**
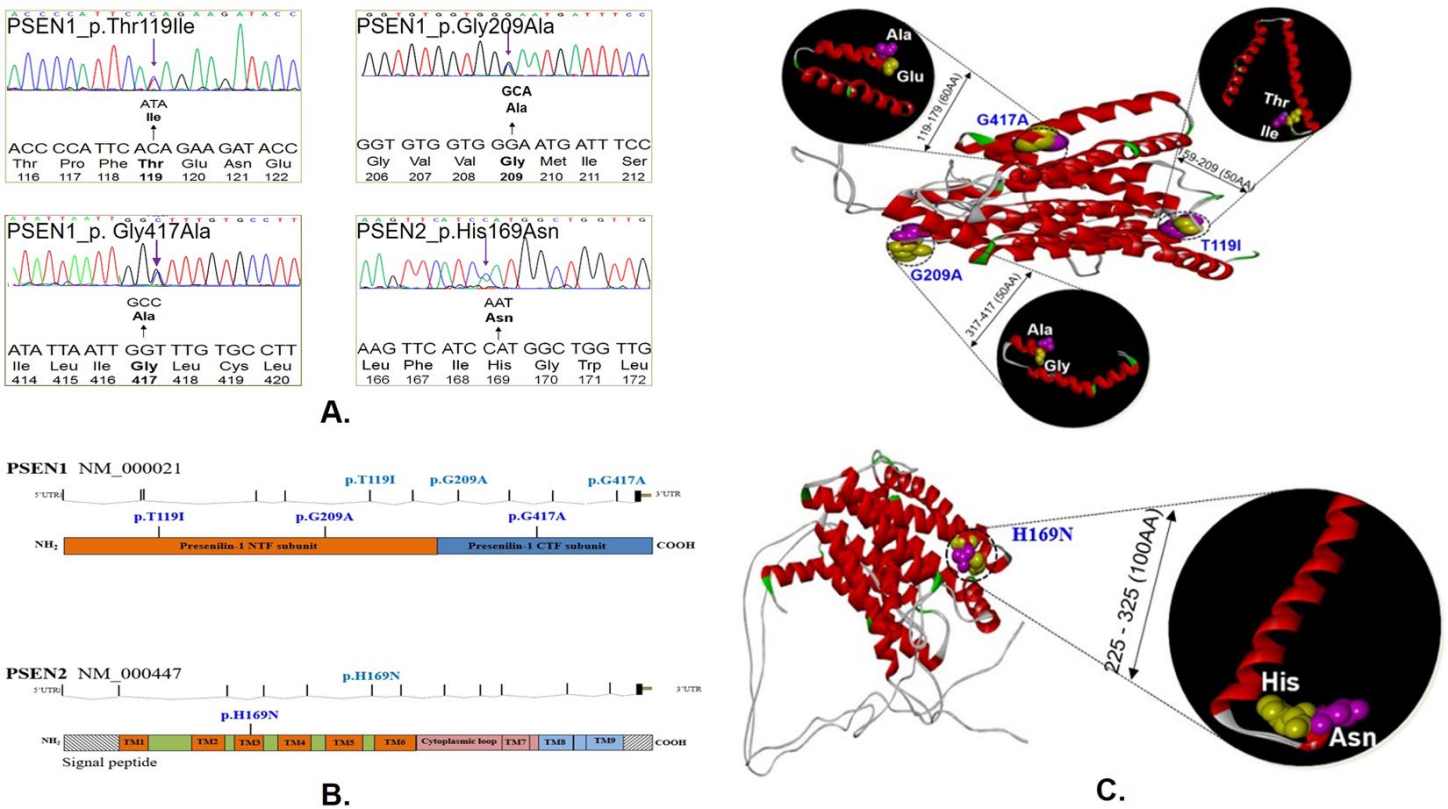
Detection of *PSEN1* and *PSEN2* genes mutations in early-onset Alzheimer’s disease. (**A**) DNA sequencing data of the four mutations found in this study. (**B**) Location of *PSEN1* and *PSEN2* mutations in the genomic DNAs and protein. (**C**) Possible protein structure changes, associated with the *PSEN1* and *PSEN2* mutations, where the differences of normal and mutant these proteins have been highlighted in the black circle, and normal was labeled with yellow, while mutant was labeled with pink. In the protein structure, red means the alpha helices or TM regions, and grey is for the hydrophilic loops (HL) while green means the kinks in the helices or loops.

In *PSEN1*, three mutations, p.Thr119Ile (c.356 C > T), p.Gly209Ala (c.626 G > C), and, p.Gly417Ala (c.1250 G > C), were identified (Table 1, Fig. 1). *PSEN1* p.Thr119Ile was identified in an EOAD patient in whom disease onset occurred at 64 years. The first symptoms appeared in 2012 and included memory impairment and language problems. Mini-Mental State Examination (MMSE)and Clinical Dementia Rating (CDR) scores were 28 and 0.5, respectively, and the patient was definitely diagnosed with AD. Analysis by 18 F-fludeoxyglucose and positron emission tomography (FDG-PET) revealed decreased metabolism in the bilateral, parietal, and temporal cortices. *PSEN1* Thr119Ile is located in a conservative HL-I, and a similar pathogenic mutation, Thr116Ile, was found in the same loop. Thus, Thr119Ile may present similar effects as the Thr116Ile mutation^7^. To validate the NGS data, a positive sample was used for screening^35^, in which a novel missense mutation was reported, p.Gly209Ala (c.626 G > C). The G-to-A substitution at nucleotide 209 (Gly209Ala) in *PSEN1* gene was observed in 54-year-old right-handed female patient who presented a 12-year history of progressive memory decline. Interestingly, *PSEN1* Gly417Ala was found in a 37-year-old male patient diagnosed with symptoms that overlap between AD and Parkinsonism, followed by progressive language disturbance and behavioral changes with Parkinsonism. *PSEN1* Gly417Ala is in the transmembrane domain-VIII and may result in increased stress inside the transmembrane helix (TM), as alanine is more hydrophobic than glycine. Additionally, this mutation may impair splicing of the PSEN1 transcriptome, as it is adjacent to the splice site^6^. In *PSEN2*, a previously reported^12,36^ mutation, p.His169Asn (c.505 C > A), was found in a 63-years-old-female patient with EOAD who developed cognitive decline, memory problems, and language impairment beginning in 2009. Korean Mini Mental State Examination (K-MMSE) score was 0 and CDR score was 3, revealing that the patient had severe dementia. Initially, *PSEN2* His169Asn was identified in two unrelated Chinese individuals, one affected by familial LOAD (age at onset of 62 years) and the other by apparently sporadic FTD (age at onset of 68 years).

**Table 1:** Genetic, clinical and pathological characteristics of definite AD cases carrying pathogenic mutations or risk variants of *PSEN1* and *PSEN2*.

Genes	DNA change	Protein change	AOO	Gender	ApoE	ExAC frequency	PolyPhen2 HumDiv	Sift score	Provean	Family history	Clinical features
*PSEN1*	c.356 C > T (Exon 5)	p.Thr119Ile	64	F	ε33	Novel	0.9 (D)	0.06 (T)	−2.37 (N)	Unknown	EOAD
	c.626 G > C (Exon 7)	p.Gly209Ala	54	F	ε33	Novel	1 (D)	0 (D)	−5.67 (D)	Probable positive	AD, depression
	c.1250 G > C (Exon 12)	p.Gly417Ala	37	M	ε33	Novel	0.99 (D)	0 (D)	−5.33 (D)	Unknown	AD with Parkinsonism
*PSEN2*	c.505 C > A (Exon 6)	p.His169Asn	59	F	ε33	0.0001648	0.925 (D)	0.04 (D)	−6.33 (D)	Unknown	Left dominant AD

Abbreviations: AD, Alzheimer’s disease; PSEN1, presenilin 1; PSEN2, presenilin 2; AOO, age of onset; ApoE, Apolipoprotein E; ExAC, the Exome Aggregation Consortium; EOAD, early-onset Alzheimer disease; F, female; M, male; D, damaging; T, tolerant; N, neutral.

### Mutations in genes-associated with LOAD

Given that three EOAD-related genes (*APP*, *PSEN1*, and *PSEN2*) contained no mutations, we identified 67 non-synonymous variants in the LOAD risk factor genes. Possible pathogenic mutations or risk variants were found in *S100A9*, *CR1*, *BIN1*, *CLU*, *CTNNA3*, *DNMBP*, *SORL1*, *BACE1*, *GAB2*, *LPR6*, *ADAM10*, *ABCA7*, and *CD33* (Table S2). No missense mutations were identified in either *TREM2* or in *PICALM* among the cohort. Of these, we analyzed the 32 rare coding variants (minor allele frequency <1%). Remarkably, recent studies confirmed the role of rare variants in *SORL1* and *ABCA7* in both EOAD and LOAD risk^37–41^ which have been shown to increase Aβ40 and/or Aβ42 secretion when expressed *in vitro*^37^. In this study, 10 missense variants were identified in *SORL1*. Remarkably, approximately 33% (22/66) of patients in the cohort carried a common variant (rs2298813-p.Ala528Thr), which was demonstrated to increase Aβ42 secretion in AD cases by altering Aβ levels and interfering with APP trafficking^37,38^. Additionally, we identified a total of 24 missense variants in *ABCA7* in the Korean AD cohort referred for medical genetic testing. Based on *in silico* functional analysis, many variants were probably predicted as damaging. Interestingly, a stop-gain mutation in *ABCA7* (p.Trp1214Ter (rs201060968) was suggested as evidence of loss-of-function mutations that may be related to the pathogenetic mechanism of AD^42^, which also was found in this study from a patient with EOAD. Additionally, one frameshift mutation of 7 base pairs (p.Asp540fs), leading to a premature stop codon, was highly significant and should be further examined. Notably, because clusterin (*CLU*) was suggested as the second highest genetic risk factor for AD, the novel variant in *CLU* Arg127His was identified in an EOAD patient. These findings may support the important roles of *CLU* mutations in the AD pathogenesis. In summary, we found several probable significant novel mutations, and these variants commonly associated with LOAD susceptibility genes may have a biological function related to AD. These findings suggested that these LOAD gene defects played important roles in the pathogenesis of the AD cohort, which may explain the missing genetic etiology of EOAD.

### Mutations in neurodegeneration-associated genes

The overlapping clinical and neuropathologic features between AD and other neurodegenerative dementias (FTD, corticobasal degeneration [CBD], progressive supranuclear palsy [PSP], and CJD) may result misdiagnosis in 17–30% of AD cases^43^. Additionally, because rare high-penetrant mutations in *APP*, *PSEN1*, and *PSEN2* explained only a small fraction of EOAD families, leaving a large group of autosomal dominant pedigrees genetically unexplained. To fill this gap in knowledge, we explored the frequencies and spectrum of mutations in genes previously implicated in EOAD by NGS of a cohort of patients with pathologically confirmed EOAD. We identified 70 non-synonymous variants in the patients in 21 dementia-related genes, namely *SIGMAR1*, *FUS*, *GRN*, *MAPT*, *ALS2*, *TAF15*, *FIG*. *4*, *OPTN*, *DAO*, *HNRNPA1*, *PINK1*, *PARK7*, *SNCA*, *ATP13A2*, *PARK2*, *LRRK2*, *SPAST*, *SPG11*, *CSF1R*, *NOTCH3*, and *PRNP*. In contrast, no missense mutation were found in *TARDBP*, *CHMP2B*, *VCP*, *UBQLN2*, *SOD1*, *ANG*, *VAPB*, *SQSTM1*, *GBA*, or *CYP7B1* (Table S3). Based on these results, the underlying genetics of AD may be more complex and heterogeneous than previously thought, as many potential variants are present in genes associated with other forms of neurodegeneration as PD, FTD, ALS, and also Prion diseases. This may support previous findings demonstrating the existence of pathogenic mutations in a wide spectrum of clinical neurological disorders including AD, FTD, ALS, and PSP in AD and all types of dementia^44^. Remarkably, two known heterozygosity mutations, Gly219Lys and p.Met129Val in *PRNP*, were detected in these EOAD cases at a high frequency in this study. Approximately 3.3% (1/67) of AD patients showed heterozygosity in codon 129 (Met/Val alleles), whereas codon 219 (Glu/Lys alleles) was also detected in 10.9% (7/67) of cases, suggesting whether these common variants are benign polymorphisms (Table S4). A possible risk factor for CJD, *PRNP* p.Met232Arg, was also identified in an EOAD case, suggesting that *PRNP* mutations were present in Korean patients with AD. Although rare cases of co-occurrence of cerebral autosomal dominant arteriopathy with subcortical infarcts and leukoencephalopathy (CADASIL) and AD have been described^45–47^, identifying the proteins involved in the pathologic process of both diseases would validate the results of our cohort study. Interestingly, 8 genetic variants were found in *NOTCH3*, but none were found to alter cysteine residues in the protein. In contrast, significant pathological overlap was found between FTD and AD for two *GRN* mutations, Ser40Asn and Arg564Cys in exon 1 and exon 12, respectively. Arg564Cys was reported as a mutation with unclear function, but was suggested to be associated with AD^48^. Our findings suggested that several mutations in *GRN* may be risk factors for AD, and pathological and genetic overlap may be possible between these disorders^49^. We cannot exclude the possibility that some of these variants of unknown significance will be reclassified in the future based on putative functional and/or genetic arguments or may confer an increased risk for developing AD. These data will help advance our understanding of dementia-related genes in Korean and East Asian populations diagnosed with AD.

### *In silico* gene/protein functional interaction network

**Figure 2:**
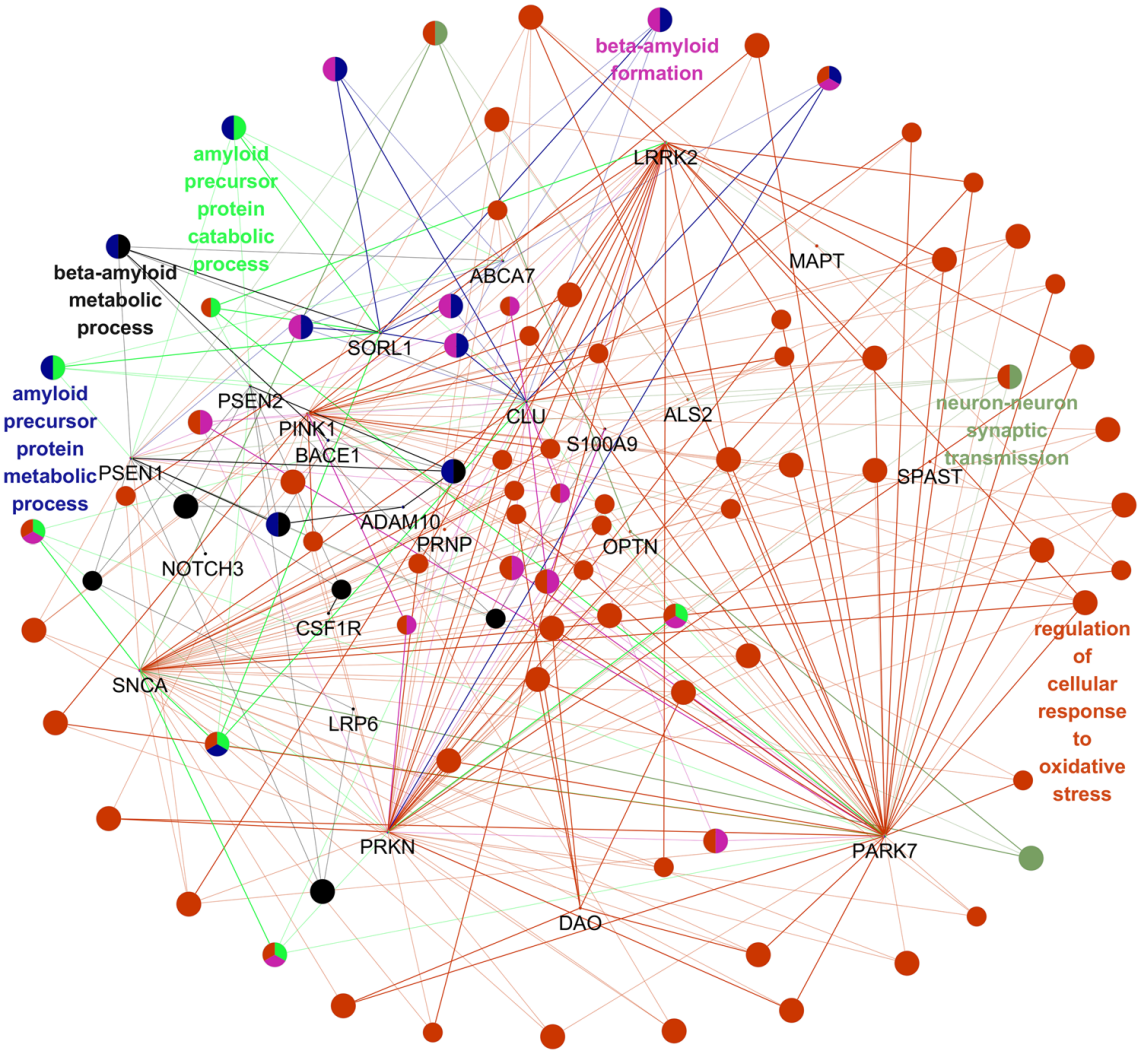
Modular partitions of the risk genes were obtained by network decomposition with the ClueGo algorithm. Gene nodes were sized by connectivity and partitioned to Modules 1–10. Potential pathways which could play a role in amyloid mechanism, such as amyloid beta formation, APP metabolism/catabolism are displayed.

The understanding of the molecular mechanisms underlying AD remains incomplete. Because AD is considered as a complex disease thought to result from the interactions of many genes and environmental factors, each gene may have only a small effect on the disease. Most previous studies focused on candidate genes and studied each gene individually, either evaluating one marker at a time or forming haplotypes over multiple neighboring loci in and around one gene. In addition, the effects of genes on phenotype result from physical interactions in gene regulatory networks and biochemical pathways in an individual. The multifactorial nature of this complex disease suggests that gene–gene interactions within biological networks may be evaluated by Gene Ontology^50^. Given that many mutations have been identified in the gene panel from the EOAD cohort, the roles of these genes and their interactions in AD remain unclear. Thus, we conducted interaction network analysis to assess 35 gene–gene (Tables S2 and S3) interactions which may be associated with AD using ClueGo. As a result, the major biological themes linked to these genes were revealed by function and biochemical pathway enrichment analysis, and relationships between pathways were also observed, suggesting involvement of the Aβ metabolic process (Fig. 2). ClueGo analysis revealed a complex network of genes that may play a role in diverse mechanisms. *PSEN1* and *PSEN2* may be involved in amyloid mechanisms, such as amyloid beta formation and APP metabolism/catabolism. Other genes potentially also play a direct or indirect role in amyloid metabolism, including *SORL1*, *ABCA7*, *CLU*, *SNCA*, and *LRRK2*. Several genes may be involved in other mechanisms, such as by regulating cellular responses to oxidative stress or synaptic transmission (*PARK7*, *SPAST*, *MAPT*, *ALS2*, *DAO*, *PARK2*, *PARK7*, and *PRNP*). However, many of these interactions require further investigation. Further studies of these pathways may increase the understanding of the patho-mechanisms related to the mutations. These results may lead to improved therapeutics targeting with Aβ accumulation as the dominant factor.

## Discussion

Recent studies demonstrated that mutations in AD-related Mendelian genes, including *APP*, *PSEN1*, and *PSEN2*, cause, contribute to, and modify the risk of AD^51,52^. Due to the clinical and pathologic overlap between AD and other neurodegenerative diseases, a expanding genetic contribution toward AD risk by genes involved in the diseases would be considered for some time now. However, to demonstrate that the variants contribute to disease at a genome-wide level requires statistical analysis of large cohorts and well-characterized populations. The frequency of rare mutations in non-Mendelian genes should also be determined, as these variants may contribute to disease through different mechanisms, such as through the impairment in amyloid metabolism, inflammatory pathways, or lipid metabolism. Because of the phenotypic similarity of patients, extensive genetic screening should be performed for those diagnosed with EOAD. AD and FTD may show similar symptoms and pathology, and several mutations in *GRN* and *MAPT* have been observed among patients with AD. Additionally, mutations in PD-causing genes have frequently been detected in EOAD, suggesting that these variants contribute to AD onset^53^.

In this study, we examined whether the significant phenotypic overlap between family/sporadic EOAD and other neurodegenerative diseases (PD, FTD, ALS, and CJD) could be explained by the common genetic background of patients. Recently, 210 familial AD-associated mutations (www.molgen.ua.ac.be/ADMutations) were linked to *PSEN1*, the catalytic subunit of the γ-secretase complex^54^, most of which lead to an increased proportion of Aβ42^55^. These mutations may be associated with gain-of function or loss-of function effects. Previously, the estimated mutation frequencies in an EOAD patient cohort for the three genes were <1% for *APP*, 6% for *PSEN1*, and 1% for *PSEN2*^18^. Thus, we developed a panel of 50 genes to evaluate whether the rare coding variability of these genes is responsible for a high proportion of EOAD risk^19^. In our EOAD cohort, we identified three rare variants of *PSEN1* (p.Thr119Ile, p.Gly209Ala, and p.Gly417Ala) and a rare known variant in *PSEN2* (p. His169Asn). A novel mutation in exon 5 of presenilin-1 (Thr119Ile) in a Korean patient with EOAD who showed memory decline at 64 years of age followed by memory impairment, language problems, and personality changes. This mutation was not found in any public databases, indicating that the polymorphism is rare. Because isoleucine may disrupt helix structures, substitution of threonine for isoleucine at codon 119 may have significantly disturbed PSEN1 protein functions. This hypothesis is supported by the results of *in silico* analyses which predicted that the T119I mutation results in major helix torsion. Several threonine > isoleucine substitutions, identified in the conservative hydrophilic loop-I of PSEN1, were associated with aggressive AD phenotype. Additionally, *PSEN1* p.Gly209Ala is in the transmembrane–IV region of the PS1 protein, where several other pathogenic variants were reported at the same residue, including Gly209Arg, Gly209Glu, and Gly209Val (http://www.molgen.ua.ac.be/ADMutations/). This may be a pathogenic variant linked to a related phenotype in EOAD and was suggested as a possible novel pathogenic variant in *PSEN1* during structural prediction using the *PSEN1* mutation. Remarkably, a known pathogenic mutation in exon 6 of *PSEN2* was identified in a Korean patient with EOAD who showed memory decline at 59 years of age, followed by memory impairment, language problems, and personality changes. Previously, this mutation was reported in a patient with familial late-onset AD with progressive memory loss beginning at the age of 68 years and in 1 sporadic early-onset patient with FTD with gradual onset of symptoms starting at 62 years of age^36^. This residue is in exon 6, transmembrane domain III, which is conserved in *PSEN1* His169, where variants (His163Pro, His163Tyr, and His163Arg) have been described as pathogenic mutations (http://www.alzforum.org)^12^. In total, less than 6% of patients with EOAD in this study carried these mutations, which explained only 5–10% of patients with EOAD as previously reported^15,17,18^.

Based on this findings, majority of early onset AD cases in the cohort remain genetically unexplained, which were also demonstrated the missing genetic etiology of EOAD (90–95%)^16^. The large number of patients with EOAD of unknown genetic background and a lack of statistical power reinforces the fact that additional causal genes need to be identified. Recent meta-analysis of GWASs identified at least 22 genes implicated in LOAD as described above, which may play a role in AD pathogenesis, although their functional role and significance remain unclear^15^. In this NGS study, several novel and known AD-related mutations were found in the cohort of 67 patients with AD, which may be associated with neurodegeneration. We also identified several mutations in other genes that cause neurodegenerative disease (such as FTD, PD, prion, or ALS). Although numerous studies have identified different genetic risk factors, including the ε4 allele of *APOE*, genetic variants have not been integrated with genetic epidemiology to quantify the age of AD onset. More recently, a study developed a polygenic hazard score for quantifying individual differences in the age-specific genetic risk for AD and found that the polygenic architecture plays an important role in modifying the AD risk beyond APOE^15^. In this study, most patients carried several validated variants within the panel of 50 genes. Our results revealed important additional variants associated with other genes; *APP*, *PSEN1*, *PSEN2*, and *APOE* genetic variants were not shown to contribute to the disease etiology. Therefore, it may be difficult to identify genetic variants associated with the disease mechanism of AD and were found in more than two patients. Given the flexibility of our genetic findings, our results are useful for investigating whether a combination of common and rare genetic variants along with clinical, cognitive, and imaging biomarkers are useful for diagnosing AD onset.

In contrast, ClueGo pathway analysis revealed complex interactions between genes. In addition to *PSEN1* and *PSEN2*, several additional AD-risk genes may play a role in amyloid peptide formation and metabolism, such as *SORL1*, *ADAM10*, *ABCA7*, and *CLU*^56^. Additionally, the ClueGo interaction also suggested that other neurodegenerative disease-causing genes impact amyloid-associated pathways, such as *LRRK2*, *PARK7*, or *SNCA*. *LRRK2* mutants were associated with reduced Aβ levels in patients with CSF of PD^57^. *LRRK2* was suggested as potential risk factor for AD^58^. Mutated LRRK2 protein may enhance the phosphorylation of the intracellular domain of APP, resulting in neurotoxicity^59^. *SNCA* may also impact AD onset, as the interaction of Aβ peptides and α-synuclein interaction may result in neurotoxicity through enhanced oxidative stress, impaired calcium metabolism, and abnormal mitochondrial pathways^60^. PARK7 or DJ1 protein may impact AD onset, as their expression levels were elevated in cases of oxidative stress^61^. Additional gene interactions were observed in the response to oxidative stress. Oxidative stress has been suggested to play a significant role in neurodegeneration and the onset of different neurodegenerative diseases, including in AD.

There were some limitations to this study. For example, we could not screen our patients for copy number variants (CNVs) or repetitive element expansions, such as duplication in *APP* or *PRNP* insertions and deletions^62^. We also could not screen for the G4C2 repeat expansion of the *c9orf72* promoter region, which was established as causative factor for FTD and ALS. However, abnormal *c9orf72* repeat expansions may be rare in Asian (Korean, Japanese) populations^13^. Additionally, we could not perform *in vitro* cell studies to verify the importance of novel mutations in AD risk genes^63^. Further, family members of patients refused genetic testing, and segregation of rare mutations could not be screened.

Our study confirmed that mutations in *APP*, *PSEN1*, and *PSEN2* are relatively rare among patients with EOAD^14,53^. Rare missense mutations were also found in additional causative genes, such as *GRN*, *MAPT*, *LRRK2*, *NOTCH3*, or *PRNP*, which may contribute to neurodegeneration^64^. We also found several mutations, including novel mutations in AD risk factor genes, such as *SORL1*, *ABCA7*, or *CD33*. Other genes may play a role in AD onset but were not included in our gene panel. We plan to perform whole exome sequencing (WES) of patients in whom no mutations were found in *APP*, *PSEN1*, or *PSEN2*. Recently, several possible candidate genes have been identified and suggested to be involved in neurodegeneration. Whole exome sequencing studies may be helpful for additional genetic profiling of patients with AD and dementia and to improve diagnosis as well as broaden the knowledge of the possible genes and pathways associated with neurodegeneration^62^.

Renewed interest in EOAD in the NGS era may improve the knowledge of the molecular and cellular mechanisms that ultimately lead to AD. In-depth genetic characterization conducted by systematic screening of dementia-causing genes, will allow for patient stratification in more homogenous groups, leading to the selection of unexplained EOAD in both familial and sporadic patients that can be further analyzed. This may be accomplished through gene identification studies or trials for biomarker selection or compound testing, providing a foundation for personalized medicine. We cannot exclude the possibility that some of these variants of unknown significance will be reclassified in the future based on putative functional and/or genetic arguments or may confer an increased risk for developing AD. Additional studies will advance our understanding of dementia-related genes in Korean and East Asian subjects diagnosed with AD.

## Materials and Methods

### Case selection

The average age of EOAD diagnosis was 58.1 years (n = 67). All patients were diagnosed as either definite or probable AD according to National Institute of Neurological and Communicative Disorders and Stroke and the Alzheimer’s disease and Related Disorders Association (NINCDS-ADRDA) and Consortium to Establish a Registry for Alzheimer’s Disease (CERAD) guidelines^65^. The family history of patients was also checked. A positive family history means that the patient had at least one family member, affected with dementia or some type of neurodegenerative disease. However, we could not verify the family history because all family members and relatives declined genetic testing.

DNA was extracted from blood samples using the GeneAll blood kit (Seoul, Republic of Korea) following the manufacturer’s protocol. DNA quality and quantity were assessed by gel electrophoresis and with a NanoDrop 3300 spectrometer, respectively. All samples were checked for the APOE genotype using the EzWay™ Direct PCR method (Komabiotech, Korea) to detect the APOE ɛ2, ɛ3, and ɛ4 alleles (Fig. S1 and Table S1**)**.

As normal controls, we used the whole genome sequencing dataset of 622 unaffected individuals from the Korean Reference Genome Database (KRGDB; http://coda.nih.go.kr/coda/KRGDB/index.jsp). We also checked all variants against larger reference databases, including the Exome Aggregation Consortium (ExAC; http://exac.broadinstitute.org/) and 1000Genomes database (http://www.internationalgenome.org/). As an internal positive control, one previously reported sample with a heterozygous missense mutation (PSEN1 G209A) was included^35^.

### Panel design

**Figure 3:**
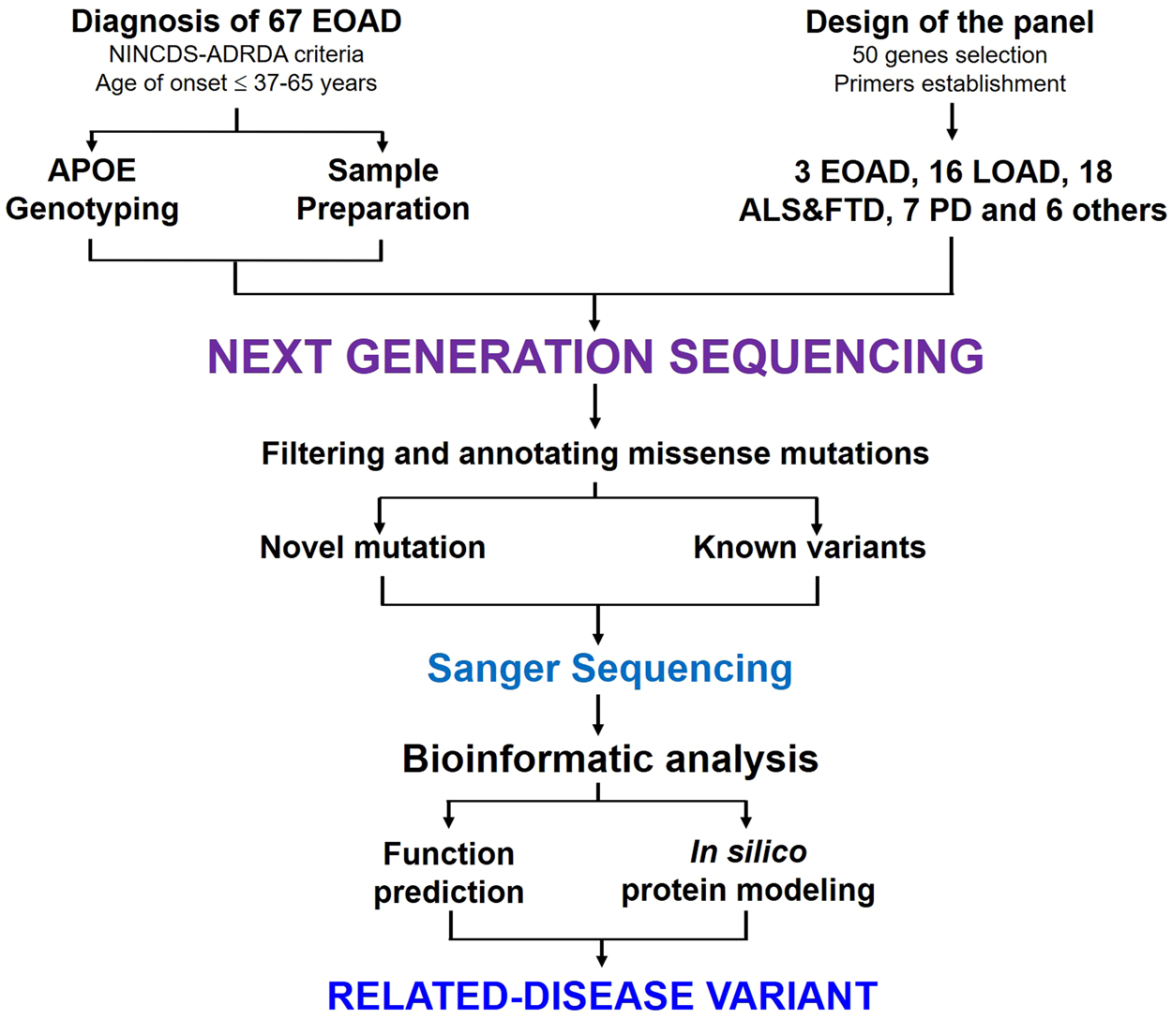
The NGS strategy for identifying variants in AD patients. The flow chart illustrated the main steps in the working procedure from the analysis of the patient sample to the identification of the mutations.

Fifty genes (Table 2**)** comprising 796 exons were ultimately selected for the targeted sequencing panel as previously reported^19^. The following candidate genes were evaluated: (1) previously reported as disease-causing mutations in patients with AD and (2) other risk factor genes of neurodegenerative diseases. A total of 876 pairs of primers were designed for the 796 exons of 50 genes and their surrounding regions. Briefly, 67 patients with EOAD were analyzed by NGS, following the schematic diagram shown in Fig. 3.

**Table 2:** List of 50 genes where causative or probably causative variants were reported to cause early-onset dementia.

Disease categories	No. of genes	Candidate genes selection
Alzheimer’s disease	19	*APP*, *PSEN1*, *PSEN2*, *S100A9*, *CR1*, *BIN1*, *TREM2*, *CLU*, *CTNNA3*, *DNMBP*, *SORL1*, *BACE1*, *PICALM*, *GAB2*, *LPR6*, *ADAM10*, *ABCA7*, *CD33*, *TOMM40*.
Amyotrophic Lateral Sclerosis (ALS) & Frontotemporal dementia (FTD)	18	*TDP43*, *CHMP2B*, *SIGMAR1*, *VCP*, *FUS*, *GRN*, *MAPT*, *UBQLN2*, *ALS2*, *TAF15*, *FIG*. *4*, *OPTN*, *DAO*, *HNRNPA1*, *SOD1*, *ANG*, *VAPB*, *SQSTM1*.
Dementia with Lewy Bodies	7	*PINK1*, *PARK7*, *PARK9*, *GBA*, *SNCA*, *PARK2*, *LRRK2*.
Other neurodegenerative diseases	6	*SPAST*, *CYP7B1*, *SPG11*, *CSF1R*, *NOTCH3*, *PRNP*.

Abbreviations: APP, amyloid precursor protein; PSEN1, presenilin 1; PSEN2, presenilin 2; S100A9, S100 calcium binding protein A9; CR1, complement receptor 1; BIN1, Bridging integrator 1; TREM2, triggering receptor expressed on myeloid cells 2; CLU, clusterin; CTNNA3, catenin alpha 3; DNMBP, dynamin-binding protein; SORL1, sortilin-related receptor; BACE1, Beta-secretase 1; PICALM, Phosphatidylinositolbinding clathrin assembly protein; GAB2, GRB2-associated binding protein 2; LPR6, Low-density lipoprotein receptor-related protein 6; ADAM10, A disintegrin and metalloprotease 10; ABCA7, ATP-binding cassette transporter A7; CD33, cluster of differentiation 33; TOMM40, translocase of outer mitochondrial membrane 40; TDP43, transactive response DNA binding protein 43 kDa; CHMP2B, charged multivesicular body protein 2B; SIGMAR1, sigma non-opioid intracellular receptor 1; VCP, valosin-containing protein; FUS, fused in sarcoma; GRN, progranulin; MAPT, microtubule associated protein tau; UBQLN2, ubiquilin 2; ALS2, amyotrophic lateral sclerosis 2; TAF15, TATA-box binding protein associated factor 15; FIG. 4, FIG. 4 phosphoinositide 5-phosphatase; OPTN, optineurin; DAO, D-amino acid oxidase; HNRNPA1, heterogeneous nuclear ribonucleoprotein A1; SOD1, Superoxide dismutase 1; ANG, Angiogenin precursor; VAPB, Vesicle-associated membrane protein-associated protein B; SQSTM1, Domain-specific mutations in sequestosome 1; PINK1, PTEN-induced kinase 1; PARK7, Parkinsonism associated deglycase 7; PARK9, Parkinson disease (autosomal recessive) 9; GBA, glucocerebrosidase; SNCA, Alpha-synuclein; PARK2, Parkinson disease associated gene 2; LRRK2, Leucine-rich repeat kinase 2; SPAST, spastin; CYP7B1, cytochrome P450 7B1; SPG11, spastic paraplegia 11; CSF1R, colony stimulating factor 1 receptor; NOTCH3, Neurogenic locus notch homolog protein 3; PRNP, prion protein.

### Ion Torrent PGM sequencing and data processing

NGS was performed in an Ion Torrent PGM system by the Theragen Etex Bio Institute (Seoul, Republic of Korea, http://www.theragenetex.com/). Fragment libraries were constructed by DNA fragmentation, and 20 ng of DNA were used for multiplex PCR of a panel covering 50 genes, where causative or probably causative variants were reported as shown in Table 2 (Ion AmpliSeq Customized Panel, Life Technologies, Carlsbad, CA, USA). Barcode and adaptor ligation and library amplification were performed using the Ion DNA Barcoding kit (Life Technologies, Grand Island, NY, USA) according to the manufacturer’s instructions. The size distribution of the DNA fragments was analyzed on the Agilent Bioanalyzer using the High Sensitivity Kit (Agilent Technologies, Santa Clara, CA, USA). Template preparation, emulsion PCR, and Ion Sphere particle (ISP) enrichment were performed using the Ion Xpress Template kit (Life Technologies, Grand Island, NY, USA) according to the manufacturer’s instructions. The ion sphere particles were loaded onto a P1 chip and sequenced with an Ion P1 sequencing 200 kit (Life Technologies, Grand Island, NY, USA).

Data analyses, including alignment to the hg19 human reference genome and variant calling, were performed using Torrent Suite Software v.4.4.3 (Life Technologies). Variants were filtered by Bam-Utils v1.0.2. Filtered variants were annotated using SnpEff v4.2. Additionally, Integrative Genome Viewer (IGV) software (http://software.broadinstitute.org/igv/) was used for mutation analysis. The joint variant calling file (VCF) was annotated with refGene gene regions, single-nucleotide polymorphism (SNP) effects, and functional effect prediction tools, as well as Exome Variant Server (EVS) and 1000 Genomes minor allele frequencies (MAFs) using Annovar (http://www.openbioinformatics.org/annovar/). For all mutations, the variants were interpreted using the Human Gene Mutation Database (HGMD, www.hgmd.cf.ac.uk), AD&FTD (www.molgen.ua.ac.be/admutations/), and AlzForum (alzforum.org/mutations) databases and by literature searches.

### Verification of mutations by Sanger sequencing

To confirm the presence of mutations, automated Sanger sequencing reactions were performed by BioNeer, Inc. (Daejeon, Korea, http://eng.bioneer.com/home.aspx) using previously reported primer sets^19^. Prior to sequencing, PCR products were purified with the GeneAll PCR protocol kit (Seoul, Korea) following the manufacturer’s protocol. Big Dye Terminator Cyclic sequencing was performed on an ABI 3730XL DNA Analyzer. Sequencing text data were aligned by NCBI Blast (http://blast.ncbi.nlm.nih.gov/Blast.cgi), and chromatograms were screened with DNA BASER (http://www.dnabaser.com) software. Mutations and sequence variants were identified by the NCBI Gene (http://www.ncbi.nlm.nih.gov/gene) and UniProt (http://www.uniprot.org) databases.

### Gene functional interaction network and *in-silico* protein modeling

In this study, 50 genes and their ontology were further analyzed to evaluate their functional influences on AD metabolic pathways by ClueGO v2.0.5. This tool visualized non-redundant biological networks of large clusters of genes, which were grouped into functional networks by statistical evaluation with respect to existing annotations in Gene Ontology^50^.

These protein change predictions were determined using SIFT (http://sift.jcvi.org/) and PolyPhen-2 (http://genetics.bwh.harvard.edu/pph2). PolyPhen2 uses a clustering algorithm and performs multiple alignment to select sequences. The algorithm uses eight sequence-based and three structure-based features for prediction, which are based on comparison of wild-type and mutant proteins. Two types of datasets can be used, HumDiv and HumVar data. HumDiv data is used to determine the role of rare alleles in complex disease phenotypes and natural selection. Alleles that may be less damaging should also be treated as possibly pathogenic. HumVar data is used to diagnose Mendelian disorders and requires the differentiation of highly damaging mutations from less damaging variants. SIFT uses different protein databases, such as SWISS-PROT, SWISS-PROT/TrEMBL, or protein databases of NCBI to calculate the possibility of the pathogenic nature of mutations by comparing mutant and normal alleles. This program scores amino acid substitutions; when the values are greater or less than 0.05, mutations can be defined as deleterious or tolerated, respectively. Additionally, PROVEAN (http://provean.jcvi.org/index.php) was used to determine the nature of mutations as benign or possible damaging.

Furthermore, some significant damaging missense mutations were further probed to determine the structures of normal and mutant protein changes by the RaptorX web server (http://raptorx.uchicago.edu/), a protein structure prediction server, using amino acid sequences. RaptorX uses a few measures including P-value, score, un-normalized global distance test (uGDT), and global distance test (GDT), uSeqID, and SeqID to evaluate the quality of a predicted 3D structure model. Next, Discovery Studio 3.5 Visualizer from Accelrys was used to display superimposed images.

### Ethical approval

The current project received ethics approval from the Seoul National University College of Medicine in Seoul National Bundang Hospital (SNUH) and written informed consent was obtained from all participants according to the requirements of the Seoul National Bundang Human Research Committee. All procedures involving human participants were conducted in accordance with the ethical standards of the institutional and/or national research committee and 1964 Helsinki Declaration and its later amendments.

## Supplementary information

Supplementary information
