# Supplementary material for: Genetic analyses of early-onset Alzheimer’s disease using next generation sequencing
Source: Sci Rep. 2019 Jun 10;9:8368. doi: 10.1038/s41598-019-44848-2 (PMC6557896; doi:10.1038/s41598-019-44848-2)
Supplement: Supplementary file 1 — Supplementary information [file 41598_2019_44848_MOESM1_ESM.pdf]

Supplementary information for

"Genetic analyses of early-onset Alzheimer's disease using next generation sequencing"

Vo Van Giau[1a], Eva Bagyinszky[1a], YoungSoon Yang[2], Young Chul Youn[3], Seong Soo A. An[1*], SangYun Kim[4*]

(1) Department of Bionano Technology, Gachon University

(2) Department of Neurology, Veterans Health Service Medical Center, Seoul, Korea

(3) Department of Neurology, Chung-Ang University School of Medicine, Chung-Ang University Hospital

(4) Department of Neurology, Seoul National University College of Medicine & Neurocognitive Behavior Center, Seoul National University Bundang Hospital

[a]These authors contributed equally to this research

+ Address for co-correspondence: Seong Soo A. An, PhD and SangYun Kim, MD, PhD

Seong Soo A. An, PhD
Department of Bionano Technology, Gachon Bionano Research Institute, Gachon University,
1342 Sungnam-daero, Sujung-gu, Seongnam-si, Gyeonggi-do,
461-701, South Korea
Tel: +82-31-750-8755
Fax: +82-31-750-8755
E-mail: seong.an@gmail.com

SangYun Kim, MD, PhD
Department of Neurology, Seoul National University Bundang Hospital, Seoul National University College of Medicine, 300 Gumidong, Bundang-gu, Seongnam-si, Gyeonggi-do,
463-707, South Korea
Tel: +82-31-787-7462
Fax: +82-31-719-6815
E-mail: neuroksy@snu.ac.kr

ApoE genotyping

ApoE genotyping with EzWayTMKit was performed by conventional PCR using purified DNA and direct used whole blood of 67 early onset Alzheimer's patients, the results demonstrated that homozygous and heterozygous genotypes generated allele-specific amplicons (**Figure S1** and **Table S1**). The ε3/ε4 genotype contains Cys and Arg at codon 112 and Arg at codon 158, produced 113 bp product and 308 bp and 444 bp products as shown on lane 2. Also, the heterozygote ε2/ε3 genotype, which contains Cys at codon 112 and Arg at codon 158, produced a 113 bp product at Cys primers and a 253 bp and 308 bp products as revealed at lane 3. The homozygote ε4/ε4 genotype, which possesses Arg at both codons 112 and 158, formed 308 bp and 444 bp products at lane 4. Also, the homozygote ε3/ε3 genotype, which contains Cys at codon 112 and Arg at codon 158 showed at lane 5.

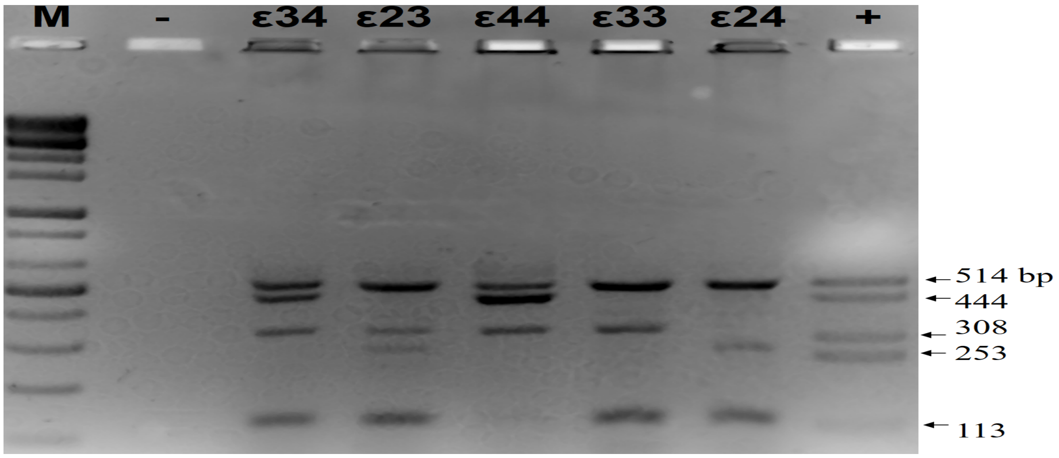

Figure S1. Gel electrophoresis of APOE genotypes following the patterns of restriction fragments

67 patients from Korean families who were diagnosed with AD were recruited for this study. Among the patients, the distribution of APOE genotypes was as follows: ε2/ε3 (n = 2; 2.9%), ε3/ε3 (n = 46; 68.7%), ε3/ε4 (n = 13; 19.4%), ε4/ε4 (n = 5; 7.4%) and ε2/ε4 (n = 1; 1.4%). Furthermore, there were significant patients have carried APOE ε3 allele, which have accounted for 61.0%, followed with 24% patients who carried at least one ApoE ε4 allele only.

Noticeably, since the carrier of two APOE ε4 alleles have a higher risk and earlier onset of AD than heterozygous subjects. In this presenting, a total of 5 AD patients with two copies of the ε4 allele (ε4/ε4) were identified.

Table s1. Genotypes and frequency of ApoE variants in the patients

**AD patients**	**ApoE allele frequencies (%)**			**ApoE genotypes % (n)**					
	ε2	ε3	ε4	ε2/ε2	ε2/ε3	ε2/ε4	ε3/ε3	ε3/ε4	ε4/ε4
N = 67	3.0	61.0	24.0	0	2.9 (2)	1.4 (1)	68.7 (46)	19.4 (13)	7.4 (5)

Table S2. Missense mutation of the risk factor genes implicated in EOAD identified by NGS genetic analysis of the cases. Genome coverage with sequencing depth 90≥%. *(NA: EOAD, early-onset Alzheimer's disease; NGS, next-generation sequencing; Not applicable; In SIFT prediction, T means tolerated, D means damaging, while in PolyPhen2, D means damaging, B means benign; N: Neutral)*

No.	Gene symbol	Accession	cDNA change	Protein change	Mutation frequency	SNP-ID	KCDC frequency	ExAC frequency	PolyPhen2 HumDiv	Sift score	Provean	Clinical significance
1	***S100A9***	NM_002965.3	c.295G>A	p.Gly99Lys	2/67	rs143906646	0.009646	0.0006330	0.002 (B)	0.7 (T)	-0.12 (N)	Probable benign variant
2	***CR1***	NM_000573.3	c.394A>C	p.Thr132Pro	1/67	rs55906048	0.001608	0.0004167	0.07 (B)	0.345 (T)	-0.87 (N)	Probable benign variant
			c.4973A>G	p.His1658Arg	19/67	rs2274567	0.204180	0.2510	0.98 (D)	0.89 (T)	-1.81 (N)	Possible risk factor for AD
			c.5573C>T	p.Thr1858Met	46/67	rs3737002	0.425241	0.2750	0.995 (D)	0.019 (D)	-0.83 (N)	Common variant, possible AD risk factor
			c.6178A>T	p.Thr2060Ser	67/67	rs4844609	1	0.9853	0.95 (D)	0.019 (D)	0.55 (N)	Common variant, possible AD risk factor
			c.6193A>G	p.Ile2065Val	21/67	rs6691117	0.220257	0.3341	0.005 (B)	1 (T)	0.49 (N)	Common variant, possible AD risk factor
			c.6830C>G	p.Pro2277Arg	20/67	rs3811381	0.191318	0.2403	0.032 (B)	0.44 (T)	-0.61 (N)	Probable benign variant

			c.7255A>G	p.Thr2419Ala	61/67	rs2296160	0.664791	0.8159	0.001 (B)	0.98 (T)	-0.10 (N)	Common variant
3	***BIN1***	NM_004305.3	c.1292C>T	p.Pro431Leu	5/67	rs141119288	0.015273	0.003413	0.99 (D)	0.07 (T)	-2.26 (N)	Probably AD risk factor
4	***CLU***	NM_001831.3	c.380G>A	p.Arg127His	1/67	NA	0.000804	0.00004944	0.56 (D)	0.003 (D)	-0.65 (N)	Probably AD risk factor
5	***CTNNA3***	XM_005269717.1	c.1787G>A	p.Ser596Asn	47/67	rs4548513	0.431672	0.4120	0.001 (B)	1 (T)	2.04 (N)	Possible risk factor for LOAD and autism
			c.1850T>C	p.Ile617Thr	2/67	NA	0.005627	0.0002645	0.928 (D)	0.001 (D)	-2.86 (D)	Unknown significance
			c.2404G>T	p.Asp802Tyr	1/67	NA	Not found	0.00005799	0.99(D)	0.001(D)	-6.18 (D)	Unknown significance
6	***DNMBP***	NM_015221.2	c.2633C>T	p.Als878Val	1/67	rs116707029	0.002412	0.00009060	0.67 (D)	0.035 (D)	-2.25 (N)	Unknown significance
			c.3154C>T	p.Arg1052Trp	1/67	rs115117818	0.002412	0.00009117	1 (D)	0.001 (D)	-5.30 (D)	Unknown significance
			c.3410G>A	p.Arg1137Gln	1/67	rs116296676	0.004019	0.0006096	0.99 (D)	0.006 (T)	-2.58 (D)	Unknown significance
			c.4138G>A	p.Glu1380Ser	1/67	rs148004684	0.003215	0.0006599	0.002 (B)	0.94 (T)	0.55 (N)	Unknown significance
			c.4239T>G	p.Cys1413Trp	28/67	rs11190305	0.261254	0.3442	0.094 (B)	0.18 (T)	-1.61 (N)	Common variant

7	***SORL1***	NM_003105.5	c.829G>C	p.Val277Leu	1/67	NA	0.003215	0.00002472	0.173 (B)	0.01 (T)	0.75 (N)	Unknown significance
			c.1528G>C	p.Glu511Arg	1/67	NA	Nor found	Nor found	0.99 (D)	0 (D)	-2.86 (D)	AD risk factor
			c.1582G>A	p.Ala528Thr	21/67	rs2298813	0.172830	0.07219	0.59 (D)	0.3 (T)	0.12 (N)	AD risk factor
			c.2586T>G	p.Asp862Glu	1/67	NA	Not found	0.0001318	0.178 (B)	0.05 (D)	0 (N)	Unknown
			c.3220C>G	p.Glu1074Gln	67/67	rs1699107	1.000000	0.9949	0 (B)	0.16 (T)	0.42 (N)	Common variant
			c.3476G>A	p.Arg1159Gln	1/67	rs527617199	Not found	0.00000864	0.62 (D)	0.45 (T)	0.12 (N)	Unknown
			c.4447A>G	p.Thr1483Ala	1/67	rs143286467	0.007235	0.001044	0.002 (D)	0.3 (T)	0.12 (N)	Unknown
			c.4570G>A	p.Glu1524Arg	3/67	rs201415809	0.001608	0.0001813	0.99 (D)	0.005 (D)	-5.12 (D)	Unknown
			c.5899A>G	p.Val1967Ile	67/67	rs1792120	0.998392	0.9953	0.003 (B)	1 (T)	0.12 (N)	Common, unknown
			c.6289G>A	p.Val2097Ile	1/67	rs74642146	0.004019	0.001203	0.9 (D)	0.16 (T)	0.16 (N)	Unknown
8	***BACE1***	NM_012104.4	c.1141C>T	p.Cys481Arg	67/67	rs539765	1.000000	0.9997	0 (B)	0.4 (T)	0.42 (N)	Common mutation

9	***GAB2***	NM_080491.2	c.404G>T	p.Glu135Val	3/67	rs76853537	0.006431	0.009359	0.006 (B)	0.5(T)	0.46 (N)	Unknown
			c.890C>T	p.Thr297Met	2/67	rs115318063	0.004823	0.0003378	0.05 (B)	0.08 (T)	-1.99 (N)	Unknown
			c.950C>T	p.Pro317Leu	2/67	rs116286425	0.032154	0.001978	0.998 (D)	0.01 (D)	-4.29 (D)	Unknown
10	***LRP6***	NM_002336.2	c.379T>A	p.Ser127Thr	3/67	rs17848270	0.040193	0.001980	0.001 (B)	0.8 (T)	-0.08 (N)	Unknown
			c.427A>G	p.Ile143V	1/67	NA	Not found	0.00000834	0.93 (D)	0.13 (T)	-0.65 (N)	Unknown
			c.2902C>T	p.R968Trp	1/67	NA	Not found	0.00000823	0.99 (D)	0.001 (D)	-5.49 (D)	Unknown
			c.3184G>A	p.Val1062Ile	67/67	rs2302685	0.922830	0.8474	0 (B)	1 (T)	0.96 (N)	Common mutation, possible AD risk factor
11	***ADAM10***	NM_001110.2	c.763A>G	p.Thr255Ala	2/67	rs147572059	0.005627	0.0007415	0.004 (B	0.88 (T)	0.36 (N)	Unknown
			c.1661A>G	p.Asp554Gly	1/67	NA	Not found	Not found	0.02 (B)	0.377 (T)	-1.32 (N)	Unknown
12	***ABCA7***	NM_019112.3	c.563A>G	p.Glu188Gly	46/67	rs3764645	0.428457	0.4838	0.06 (B)	0.67 (T)	-0.87 (N)	Unknown
			c.955A>G	p.Thr319Ala	11/67	rs3752232	0.094052	0.06039	0.142 (B)	0.446 (T)	1.03 (N)	Possible AD risk factor

			c.1184A>G	p.His395Arg	9/67	rs3764647	0.099679	0.06298	0.02 (B	1 (T)	0.11 (N)	Possible AD risk factor
			c.1384G>C	p.Val462Leu	1/67	NA	Not found	Not found	0.08 (B)	0.086 (T)	-1.92 (N)	Unknown
			c.1388G>A	p.Arg463His	18/67	rs3752233	0.163183	0.04775	0.8 (D)	0.25 (T)	-2.89 (D)	Unknown
			c.1620delC	p.Asp540fs	1/67	NA	0.0008	0.00002735	NA	NA	NA	Unknown
			c.1570C>T	p.Arg524Trp	1/67	NA	0.001608	0.0002259	0.75 (D)	0.01 (D)	-3.25 (D)	Unknown
			c.1832T>G	p.Val611Gly	1/67	NA	0.000804	0.0001286	0.97 (D)	0.004 (D)	-4.34 (D)	Unknown
			c.2153A>C	p.Asn718Thr	20/67	rs3752239	0.154341	0.07028	0.58 (D)	0.24 (T)	-5.17 (D)	Unknown
			c.2892C>G	p.Asp964Glu	2/67	rs117390715	0.008039	0.001506	1 (D)	0 (D)	-3.61 (D)	Unknown
			c.2923C>T	p.Arg975Cys	1/67	NA	0.000804	0.0001787	1 (D)	0 (D)	-7.25 (D)	Unknown
			c.3629G>A	p.Arg1210Gln	1/67	NA	NA	0.00009019	0.006 (B)	1 (T)	0.66 (N)	Unknown
			c.3641G>A	p.Trp1214*	1/67	NA	0.004823	0.0005178	NA	NA	NA	AD risk factor, stop codon

			c.4046G>A	p.Trp1349Gln	35/67	rs3745842	0.366559	0.4433	0.004 (B)	0.54 (T)	0.05 (N)	Common variant, unknown
			c.4211A>G	p.Lys1404Arg	1/66	NA	Not found	Not found	0.03 (B)	0.002 (D)	-2.14 (N)	Unknown
			c.4580G>C	p.Gly1527Ala	58/67	rs3752246	0.661576	0.8405	0 (B)	0.87 (T)	2.70 (N)	Common variant, unknown
			c.5057A>G	p.Gln1686Arg	16/67	rs4147918	0.150322	0.04785	0.004 (B)	0.23 (T)	-0.44 (N)	Common variant, unknown
			c.5069T>C	p.Ile1690Thr	2/67	rs184218896	0.017685	0.0002979	0.911 (D)	0.001 (T)	-3.74 (D)	Unknown
			c.5339G>A	p.Arg1780Qln	2/67	NA	0.001608	0.00009783	0.88 (D)	0.002 (D)	-3.63 (D)	Unknown
			c.5609G>T	p.Gly1870Val	1/67	NA	Not found	0.00000839	0.99 (D)	0 (D)	-8.31 (D)	Unknown
			c.5692C>T	p.Pro1898Ser	1/67	NA	Not found	0.00000849	0.9 (D)	0.08 (T)	-7.38 (D)	Unknown
			c.5789A>G	p.Asn1930Ser	1/67	NA	Not found	Not found	0.98 (D)	0.002 (D)	-4.27 (D)	Unknown
			c.5963G>T	p.Cys1988Phe	1/67	rs142573667	0.001608	0.0004807	1 (D)	0 (D)	-9.39 (D)	Unknown
			c.6133G>T	p.Ala2045Ser	45/67	rs4147934	0.422830	0.7317	0.051 (B)	0.96 (T)	0.06 (N)	Common mutation, unknown

			c.6633G>T	p.Phe2071Cys	2/67	NA	0.006431	0.002584	0.5 (B)	0.46 (T)	-6.84 (D)	Common mutation, unknown
13	***CD33***	NM_001772.3	c.41C>T	p.Ala14Vla	26/67	rs12459419	0.174437	0.2939	0.023 (B)	0.08 (T)	-2.12 (N)	AD risk factor
			c.205A>G	p.Arg69Gly	4/67	rs2455069	0.058682	0.3577	0.009 (B)	0.33 (T)	-2.36 (N)	Unknown
			c.594A>G	p.IleI198Met	1/67	rs200827254	0.009646	0.0008239	0.105 (B)	0.006 (T)	-0.03 (N)	Unknown
			c.880G>T	p.Val294Leu	1/67	rs2271652	0.010450	0.0008755	0.001 (B)	0.16 (T)	-1.11 (N)	Unknown

Table S3. Missense mutation of other dementia genes implicated in EOAD identified by NGS genetic analysis of the cases. Genome coverage with sequencing depth 90≥%. (*NA: Not applicable; In SIFT prediction, T means tolerated, D means damaging, while in PolyPhen2, D means damaging, B means benign)*

No.	Gene symbol	Accession	cDNA change	Protein change	Mutation frequency	SNP-ID	KCDC frequency	ExAC frequency	PolyPhen2 HumDiv	Sift score	Provean	Clinical significance
1	***PINK1***	NM_032409.2	c.755A>T	p.Glu252Val	1/67	NA	Not found	Not found	0.605 (D)	0.05 (D)	-3.91 (D)	Unknown
			c.1018G>A	p.Ala340Thr	38/67	rs3738136	0.305466	0.09211	0.001 (B)	1 (T)	1.34 (N)	Unknown
			c.1562A>C	p.Asn521Thr	47/67	rs1043424	0.377010	0.09211	0.005 (B)	0.24 (T)	-0.94 (N)	Unknown
2	***PARK7***	NM_007262.4	c.535G>A	p.Ala179Thr	1/67	rs71653622	0.002412	0.0006193	0. 001 (B)	0.56 (T)	-0.15 (N)	Unknown
3	***ATP13A2 (PARK9)***	NC_000001.11	c.317A>G	p.Glu106Arg	1/67	NA	Not found	Not found	0.008 (B)	0.47 (T)	-0.29 (N)	Unknown
			c.1499G>T	p.Arg500Leu	1/67	NA	Not found	Not found	1 (D)	0 (D)	-5.27 (D)	Unknown
			c.1817C>G	p.Pro606Arg	1/67	NA	Not found	Not found	0.93 (D)	0.025 (D)	-4.44 (D)	Unknown
4	***SNCA***	NM_001146055	c.381G>A	p.Met127Ile	1/67	rs191055637	0.007235	0.00009900	0.77 (D)	0.16 (T)	-0.57 (N)	Unknown

5	***PARK2***	NM_004562.2	c.500G>A	p.Ser167Asn	45/67	rs1801474	0.456592	0.06758	0.03 (B)	0.31 (T)	-1.45 (N)	Benign variant
			c.799T>C	p.Tyr267His	1/67	rs114696251	0.000804	0.0001995	0.99 (D)	0 (D)	-4.21 (D)	Unknown
			c.1138G>C	p.Val380Leu	4/67	rs1801582	0.044212	0.1646	0 (B)	0.37 (T)	-0.09 (N)	Benign variant
6	***LRRK2***	NM_198578.3	c.691T>C	p.Ser231Pro	1/67	rs201332859	0.000804	0.00004977	0.004 (B)	0.3 (T)	0.00 (N)	Unknown
			c.1256C>T	p.Ala419Val	2/67	rs34594498	0.015273	0.0000083	0.49 (D)	0.03 (D)	-1.18 (N)	PD risk factor
			c.1598T>C	p.Val533Ala	1/67	NA	0.000804	0.0000082	0.5 (T)	0.004 (D)	-1.89 (N)	Unknown
			c.1653C>G	p.Asn551Leu	16/67	rs7308720	0.152733	0.08607	0.005 (D)	0.972 (D)	-2.63 (D)	Possible PD risk factor
			c.2167A>G	p.Ile723Val	2/67	rs10878307	0.008842	0.05922	0.001 (B)	0.68 (T)	-0.02 (N)	Possible PD risk factor
			c.4193G>A	p.Arg1398His	14/67	rs7133914	0.136656	0.08412	0.58 (D)	0.1 (T)	-1.19 (N)	Possible PD risk factor
			c.4337C>T	p.Pro1446Leu	2/67	rs74681492	0.004823	0.0002473	1 (D)	0.008 (T)	-5.55 (D)	Possible risk factor
			c.4939T>A,	p.Ser1647Thr	29/67	rs11564148	0.260450	0.2983	0 (B)	0.95 (T)	-0.15 (N)	Possible risk factor

			c.7153G>A	p.Gly2385Arg	6/67	rs34778348	0.016077	0.1129	0.013 (B)	0.4 (T)	0.25 (N)	Possible PD risk factor
			c.7190T>C	p.Met2397Thr	43/67	rs3761863	0.418810	0.6240	0 (B)	0.466 (T)	-0.53 (N)	Possible PD risk factor
7	***SIGMAR1***	XM_005251339	c.5A>C	p.Gln2Pro	42/67	rs1800866	0.319936	0.1840	0 (B)	0.343 (T)	0.03 (N)	Possible risk factor
			c.622C>T	p.Arg208Trp	5/67	rs11559048	0.024920	0.007782	0.94 (D)	0.01 (D)	-4.42 (D)	Unknown
			c.632G>A	p.Arg211Gln	1/67	rs192644838	0.024920	0.00009934	0.57 (D)	0.29 (T)	-0.28 (N)	Unknown
8	***FUS***	NM_004960.3	c.451C>T	p.Pro151Ser	1/67	NA	Not found	0.00000823	0.007 (B)	0.03 (D)	-1.39 (N)	Unknown
9	***GRN (PGRN)***	NM_002087.2	c.119G>A	p.Ser40Asn	1/67	NA	Not found	Not found	0.75 (D)	0.015 (D)	-1.02 (N)	Probable novel mutation
			c.1690C>T	p.Arg564Cys	1/67	NA	Not found	0.00000830	0.87 (D)	0.04 (D)	-2.88 (D)	AD risk factor mutation
10	***MAPT***	NM_001203251	c.418C>T	p.Pro140Ser	2/67	rs151115928	0.007235	0.0007273	0.846 (D)	0.04 (D)	-1.00 (N)	Unknown
			c.1321T>C	p.Tyr441His	59/67	rs112757188	Not found	0.2752	0 (B)	0.97 (T)	1.23 (N)	Common mutation, unknown

11	***TAF15***	NC_000017.11	G / GGGCAGAGGA	Gly444fs	1/67	NA	Not found	0.000009964	NA	NA	NA	Unknown
12	***ALS2***	NM_020919.3	c.475G>A	p.Glu159Lys	2/67	NA	0.004019	0.006714	0.156 (B)	0.15 (T)	-1.04 (N)	Unknown
			c.1102G>A	p.Val368Met	67/67	NA	1.000000	0.9106	0.009 (B)	0.191 (T)	-0.06 (N)	Common mutation, unknown
			c.2278C>G	p.Leu760Val	1/67	NA	0.000804	0.00000834	0.99 (D)	0.14 (T)	-1.47 (N)	Unknown
			c.2953C>T	p.Leu985Phe	1/67	rs41308836	0.000804	0.00002488	0.0953 (D)	0.005 (D)	-1.86 (N)	Unknown
			c.3517G>A	p.Glu1173Leu	4/67	rs41309046	0.000804	0.002211	0.8 (D)	0.003(D)	-3.41 (D)	Unknown
			c.4892A>G	p.Tyr1631Cys	1/67	NA	Not found	Not found	0.003 (D)	0.18 (T)	-0.15 (N)	Unknown
13	***FIG4***	NM_014845.5	c.658A>G	p.Ile220Val	1/67	rs565096937	Not found	0.0002883	0.45 (T)	0.004 (T)	-0.02 (N)	Unknown
			c.1090A>T	p.Met364Leu	28/67	rs2295837	0.221061	0.07428	0.001 (B)	1 (T)	1.11 (N)	Benign
			c.1961T>C	p.Val654Ala	34/67	rs9885672	0.354502	0.2753	0 (B)	0.3 (T)	0.95 (N)	Mutation

14	***OPTN***	XM_005252335	c.293T>A	p.Met98Lys	16/67	rs11258194	0.080386	0.04519	0.04 (B)	0.92 (T)	-0.06 (N)	Involved in glaucoma
			c.964G>A	p.Glu322Lys	67/67	rs523747	0.998392	0.9973	0 (B)	1 (T)	1.93 (N)	Unknown
			c.1634G>A	p.Arg545Asn	4/67	rs75654767	0.043408	0.002902	0 (B)	1 (T)	1.52 (N)	Risk for glaucoma
15	***DAO***	NM_001917.4	c.489A>C	p.Leu163Asn	3/67	NA	Not found	Not found	0.97 (D)	0.002 (D)	-4.11 (D)	Unknown
16	***HNRNPA1***	XR_245923.1	c.631G>A	p.Gly211Ser	1/67	rs182937540	0.006431	0.001156	0.05 (B)	0.22 (T)	-2.45 (N)	Unknown
17	***SPAST***	XM_005264516	c.155A>G	p.Tyr52Cys	1/67	NA	Not found	0.00001712	0.52 (D)	0.015 (D	-0.54 (N)	Unknown
18	***SPG11***	NM_025137.3	c.437A>G	p.Asn146Gly	1/67	rs182535774	0.004019	0.0004448	0.01 (B)	0.7 (T)	-0.32 (N)	Unknown
			c.833A>G	p.Asn278Ser	10/67	NA	0.074759	0.007759	0.01 (B)	0.45 (T)	-0.85 (N)	Unknown
			c.1388T>C	p.Phe463Ser	62/67	rs3759871	0.479100	0.4657	0.009 (B)	0.341 (B)	-1.27 (N)	Unknown
			c.3206A>G	p.Gln1069Arg	1/67	NA	Not found	Not found	0.74 (D)	0.03 (D)	-1.59 (N)	Unknown
			c.6892A>G	p.Ile2298V	4/67	rs147962000	0.017685	0.001040	0.05 (B	0.6 (T)	0.11 (N)	Unknown

19	***CSF1R***	NM_005211.3	c.544G>T	p.Gly182Cys	1/67	NA	0.001608	0.00006594	0.782 (D)	0.01 (D)	-3.18 (D)	Unknown
			c.635T>A	p.Leu212Gln	1/67	NA	Not found	Not found	0.2 (D)	0.14 (T)	-0.43 (N)	Possible novel variant
			c.637G>A	p.Val213etM	1/67	NA	Not found	0.00000824	0.93 (D)	0.001 (D)	-1.58 (N)	Unknown
			c.733G>T	p.Ala245Thr	1/67	rs41338945	0.000804	0.00005022	0.008 (B)	0.6 (T)	0.37 (N)	Unknown
			c.835G>A	p.Val279Met	3/67	rs3829986	0.044212	0.002537	0.19 (B)	0.11 (T)	-0.66 (N)	Unknown
			c.1085A>G	p.His362Arg	40/67	rs10079250	0.290193	0.00009984	0.15 (B)	0.06 (T)	-3.36 (D)	Common mutation, possible cancer risk factor
			c.2806G>A	p.Gly936Ser	1/67	NA	Not found	Not found	0.78 (D)	0.01 (D)	-0.28 (N)	Possible novel variant
20	***NOTCH3***	NM_000435.2	c.709G>A	p.Val237Met	1/67	rs2285981	0.000804	0.00000844	0.25 (B)	0.001 (D)	-2.22 (N)	Unknown
			c.2208C>A	p.Asp736Glu	1/67	NA	Not found	Not found	0.97 (D)	0.004 (T)	-2.01 (N)	Unknown
			c.2320C>G	p.Pro774Ala	1/67	NA	Not found	0.00009441	0.99 (D)	0.004 (T)	-5.88 (D)	Unknown

			c.2519G>A	p.Gly840Glu	1/67	NA	0.000804	0.00004135	1 (D)	0.001 (D)	-6.91 (D)	Unknown
			c.3523C>T	p.Arg1175Trp	1/67	rs200504060	0.012058	0.001047	0.91 (D)	0.004 (D)	-5.05 (D)	Unknown
			c.3620A>G	p.Asn1207Ser	2/67	rs141418355	0.000804	0.00004172	0.99 (D)	0.002 (D)	-4.58 (D)	Unknown
			c.4348G>A	p.Ala1450Thr	2/67	NA	0.004019	0.0002155	0.61 (D)	0.11 (T)	-1.81 (N)	Unknown
			c.6678C>T	p.Ala2223Val	55/67	rs1044009	0.590032	0.7591	0.003 (B)	0.14 (T)	-1.46 (N)	Common mutation
21	***PRNP***	NM_001080121.1	c.385A>G	p.Met129Val	1/67	rs1799990	0.024920	0.3078	0.012 (B)	0.024 (D)	-0.66 (N)	Pathogenic nature complicated
			c.655G>A	p.Glu219Lys	7/67	rs1800014	0.057074	0.008728	0.003 (B)	0.035 (D)	0.00 (N)	Pathogenic nature complicated
			c.674A>G	p.Tyr225Cys	1/67	NA	Not found	0.00000830	0.98 (D)	0.001 (D)	-1.13 (N)	May be involved in CJD
			c.695T>G	p.Met232Arg	1/67	rs74315409	0.001608	0.00008521	0.707 (D)	0.001 (D)	-1.20 (N)	CJD or AD involvement

Table S4. Clinical information of the patients associated with rare mutations. (AOO: age of onset; *NA: Not applicable; M: male; F: female; AD: Alzheimer's disease; EOAD: early onset Alzheimer's disease)*

**Subject No.**	**Gender**	**AOO**	**APOE status**	**Diagnosed**	**Family History**	**Common mutation**	**Rare mutation**	**Rare mutations**
1.	M	64	ε23	AD	Negative	24	1	*ADAM10* p.Thr255Ala
2.	M	60	ε34	AD	Negative	21	1	*NOTCH3* p.Ala1927Thr
3.	F	62	ε33	AD	Negative	24	1	*NOTCH3* p.Ala1450Thr
4.	F	51	ε33	AD	Negative	25	1	*HNRNPA1* p.Gly211Ser
5.	F	52	ε34	AD	Negative	33	5	*ABCA7* p.Arg1780Gln, *GAB2* p.Gln135Val, *LRP6* p.Ser127Thr, *LRRK2* p.Gln2385Arg, *PRNP* p.Glu219Leu
6.	F	61	ε33	AD	Positive (mother)	26	1	*NOTCH3* p.Asn1207Ser
7.	M	59	ε33	AD	Negative	32	1	*ABCA7* p.Arg1790Gln
8.	M	55	ε33	EOAD	Negative	22	2	*SORL1* p.Arg1159Gln, *NOTCH3* p.Leu1518Met, *TAF15* p.Gln444fs
9.	F	53	ε33	EOAD	NA	23	4	*ALS2* p.Leu985Phe, *CSF1R* p.Val279Met, *OPTN* p.Arg545Gln, *SPG11* p.Ile2298Val

10.	M	65	ε34	EOAD	Positive (father)	30	1	*ABCA7* p.Ile1690Thr
11.	F	65	ε44	AD	NA	27	5	*PRNP* p.Glu219Lys, *GRN* p.Arg564Cys, *FUS* p.Pro151Ser, *SPG11* p.Asn278Ser, *SPAST* p.Tyr52Cys
12.	F	60	ε33	EOAD	NA	28	1	*PRNP* p.Glu219Lys
13.	F	59	ε34	AD	Negative	25	2	*ALS2* p.Glu159Lys, *LRRK2* p.Val533Ala
14.	M	59	ε33	AD	Positive (father)	24	3	*CTNNA3* p.Asp802Tyr, *ABCA7* p.Ile1690Thr, *NOTCH3* p.Asn1207Ser
15.	F	63	ε33	EOAD	NA	20	1	*PINK1* p.Glu252Val
16.	F	61	ε33	EOAD	NA	28	3	*NOTCH3* p.Gly1779Val, *CTNNA3* p.Ile617Thr, *LRRK2* p.Gly2385Arg
17.	F	62	ε33	AD	NA	25	3	*CSF1R* p.Leu212Gln, p.Val213Met, *SPG11* p.Ile2298Val
18.	M	64	ε34	EOAD	NA	22	3	*CR1* p.Thr132Pro, *SIGMAR1* p.Arg211Gln, *NOTCH3* p.Ala1450Thr
19.	F	60	ε34	AD	Positive (mother)	26	1	*LRRK2* p.Pro1446Leu
20.	M	55	ε33	AD	Negative	23	2	*ABCA7* p.Asp964Glu, p.Phe2071Cys
21.	F	54	ε33	AD	Negative	27	3	*GAB2* p.Thr297Met, *LRRK2* p.Ala419Val, *SPG11*

								p.Asn278Ser
22.	F	49	ε34	AD	Negative	28	3	*SORL1* p.Val277Leu, *LRRK2* p.Ala419Val, p.Ile1967Val, *ABCA7* p.Val611Gly
23.	F	50	ε34	EOAD	Negative	26	2	*ABCA7* p.Gly1870Val, *LRRK2* p.Gly2385Arg
24.	M	59	ε33	EOAD	Negative	19	4	*BIN1* p.Pro431Leu, *LRP6* p.Ser127Thr, *MAPT* p.Asp177Val, *ABCA7 p.Arg524Trp*
25.	F	53	ε33	EOAD	Negative	35	1	*ABCA7* p.Lys1404Arg
26.	F	65	ε33	AD	Negative	23	3	*LRRK2* p.Gly2385Arg, *ABCA7* p.Cys1988Phe, *NOTCH3* p.Pro2215Ser
27.	F	65	ε33	AD	Positive	27	4	*SNCA* p.Met127Ile, *CSF1R* p.Val279Met, *SPG11* p.Asn278Ser, GRN p.Ser40Asn
28.	M	50	ε24	EOAD	Positive	26	1	*BIN1* p.Pro431Leu
29.	F	60	ε33	AD	Positive (mother)	23	2	*OPTN* p.Arg545Gln, *SQSTM1* p.Arg415Trp
30.	M	59	ε44	EOAD	NA	22	1	*OPTN* p.Arg545Gln
31.	F	63	ε33	EOAD	Negative	26	1	*LRRK2* p.Ser231Pro
32.	M	57	ε33	AD	Negative	20	1	*LRRK2* p.Gly2385Arg
33.	F	52	ε33	EOAD	Negative	27	2	*SORL1* p.Cys481Arg, *ADAM10* p.Asn554Gly

34.	F	55	ε33	EOAD	NA	27	2	*SIGMAR1* p.Ar208Trp, *ABCA7* p.Pro1898Ser
35.	F	51	ε33	AD	Negative	23	5	*BIN1* p.Pro431Leu, *FIG4* p.Ile220Val, *ALS2* p.Glu1173Lys, *SORL1* p.Asn862Glu, *PRNP* p.Met232Arg
36.	F	48	ε33	EOAD	Negative	24	1	*SPG11* p.Asn278Ser
37.	F	59	ε33	EOAD	Positive (mother)	25	6	*CSF1R* p.Gly182Cys, *SQSTM1* p.Arg415Trp, *SORL1* p.Val2097Ile, *LRRK2* p.Gly2385Arg, *CD33* p.Ile198Met, p.Val294Leu
38.	M	57	ε34	AD	Positive (mother)	19	1	*SIGMAR1* p.Arg208Trp
39.	F	64	ε33	AD	Negative	26	1	*PSEN1* p.Thr119Ile
40.	M	64	ε34	EOAD	Negative	23	3	*BIN1* p.Pro431Leu, *ALS2* p.Glu159Lys, *SORL1* p.Gly511Arg, *PRNP* p.Glu219Lys
41.	F	64	ε33	AD	NA	25	2	*ALS2* p.Tyr1631Cys, *ADAM10* p.Thr255Ala, *PRNP* p.Glu219Lys
42.	F	53	ε34	EOAD	Negative	24	1	*SPG11* p.Ile2298Val
43.	M	53	ε44	AD	Not checked	25	3	*ABCA7* p.Asp504fs, *LRP6* p.Arg968Trp, *SPG11* p.Asn278Ser
44.	F	48	ε33	EOAD	Not	26	3	*SPG11* p.Asn278Ser, *NOTCH3* p.Asp736Glu,

					checked			*ATP13A2* p.Arg500Leu
45.	M	65	ε33	AD	Negative	24	2	*SORL1* p.Gly1524Arg, *NOTCH3* p.Gly840Glu
46.	F	59	ε33	AD	NA	28	3	*PSEN2* p.His169Asn, *BIN1* p.Pro431Leu, *LRP6* p.Ile143Val
47.	F	59	ε33	AD	Negative	22	3	*PARK7* p.Ala104Thr, *NOTCH3* p.Leu1518Met, p.Arg1175Trp
48.	F	62	ε34	AD	NA	22	1	*GAB2* p.Gly135Val
49.	F	60	ε33	AD	NA	22	1	*SPG11* p.Asp146Gly
50.	M	62	ε23	EOAD	Negative	22	1	*LRRK2* p.Pro1446Leu
51.	M	37	ε33	AD	Negative	19	6	*PSEN1* p.Gly417Ala, *NOTCH3* p.Leu1518Met, *S100A9* p.Glu99Lys, *GAB2* p.Pro317Leu, *PRNP* p.Met129Val, *ATP13A2* p.Pro606Arg
52.	F	46	ε33	AD	Negative	27	4	*PRNP* p.Glu219Lys, *ABCA7* p.Asp964Glu, p.Arg975Cys, p.Phe2071Cys
53.	M	50	ε44	AD	Negative	31	1	*SIGMAR1* p.Arg208Trp
54.	F	52	ε33	AD	NA	19	1	*MAPT* p.Pro140Ser
55.	F	54	ε33	AD	NA	25	2	*ABCA7* p.Asn1930Ser, *NOTCH3* p.Pro774Ala
56.	F	52	ε34	AD	Negative	29	NA	*NA*

57.	M	64	ε33	EOAD	NA	21	NA	*NA*
58.	M	62	ε33	EOAD	Positive (mother)	23	4	*CLU* p.Arg127His, *CSF1R* p.Gly936Ser, *ALS2* p.Leu760Val, *DAO* p.Leu160Asn
59.	F	53	ε33	EOAD	NA	20	1	*SQSTM1* p.Arg415Trp
60.	F	62	ε33	EOAD	Negative	27	2	*PRNP* p.Glu219Lys, *SPG11* p.Ile2298Val
61.	F	56	ε44	EOAD	Negative	25	2	*SIGMAR1* p.Arg208Trp, *GAB2* p.Gly135Val
62.	F	54	ε33	EOAD	Positive	27	2	*PSEN1* p.Gly209Ala, *SPG11* p.Asn278Ser
63.	F	46	ε33	MCI-AD	Negative	25	3	*PARK7* p.Ala179Thr, *SPAST* p.Ile592Lys, *MAPT* p.Pro140Ser, *PARK2* p.Tyr267His
64.	F	59	ε33	EOAD	Negative	24	2	*NOTCH3* p.Val237Met, *SIGMAR1* p.Arg208Trp
65.	F	63	ε33	AD	Negative	24	12	*CTNNA3* p.Ile617Thr
66.	M	60	ε33	AD	Negative	20	NA	*NA*
67.	M	65	ε33	AD	Negative	21	NA	*NA*
